# The Role of AMP-Activated Protein Kinase as a Potential Target of Treatment of Hepatocellular Carcinoma

**DOI:** 10.3390/cancers11050647

**Published:** 2019-05-10

**Authors:** Xue Jiang, Hor-Yue Tan, Shanshan Teng, Yau-Tuen Chan, Di Wang, Ning Wang

**Affiliations:** 1 School of Life Sciences, Jilin University, Changchun 130012, China; jiangxue16@mails.jlu.edu.cn (X.J.); tengss17@mails.jlu.edu.cn (S.T.); 2 School of Chinese Medicine, The University of Hong Kong, Hong Kong, China; hyhtan@hku.hk (H.-Y.T.); ecyt1@hku.hk (Y.-T.C.)

**Keywords:** hepatocellular carcinoma, AMPK, proliferation and survival, invasion and metastasis, cancer metabolism, AMPK activators

## Abstract

*Background*: Hepatocellular carcinoma (HCC) is the fifth most frequent cancer worldwide with a very high recurrence rate and very dismal prognosis. Diagnosis and treatment in HCC remain difficult, and the identification of new therapeutic targets is necessary for a better outcome of HCC treatment. AMP-Activated Protein Kinase (AMPK) is an essential intracellular energy sensor that plays multiple roles in cellular physiology and the pathological development of chronic diseases. Recent studies have highlighted the important regulation of AMPK in HCC. This review aims to comprehensively and critically summarize the role of AMPK in HCC. *Methods*: Original studies were retrieved from NCBI database with keywords including AMPK and HCC, which were analyzed with extensive reading. *Results*: Dysregulation of the kinase activity and expression of AMPK was observed in HCC, which was correlated with survival of the patients. Loss of AMPK in HCC cells may proceed cell cycle progression, proliferation, survival, migration, and invasion through different oncogenic molecules and pathways. *Conclusions*: We identified several AMPK activators which may possess potential anti-HCC function, and discussed the clinical perspective on the use of AMPK activators for HCC therapy.

## 1. Introduction

Hepatocellular carcinoma (HCC) is the fifth most frequent cancer all over the world and accounts for more than 85% of liver malignancies [1]. It is more prevalent in developing countries, within which China accounts for over 50% of the world’s newly diagnosed cases as well as HCC-related death annually [2,3]. Typically, HCC progression is triggered by nonalcoholic fatty liver disease (NAFLD), long-term exposure to aflatoxin and alcohol, or chronic infection with hepatitis B and C [4]. Epidemiological studies have revealed that the risk factors of HCC in developing countries are Hepatitis B virus (HBV) infection and aflatoxin exposure, while it is Hepatitis C virus (HCV) and alcohol in developed countries [5]. Primary curative treatments for patients with HCC are surgical resection and liver transplantation [6]. Due to the unfavorable hepatic condition in most of the patients with HCC, only around 30% of patients are suitable for surgery. The high recurrence rate of HCC resulted in poor prognosis even in patients with the hepatic operation. The 5-year overall survival rate of patients with HCC after surgery are less than 30% [7]. Non-surgical treatment is often ineffective, as the HCC cells are naturally resistant to most of the conventional chemotherapeutic agents [8,9]. Several targets therapeutic agents have been approved for HCC treatment, such as sorafenib, lenvatinib and regorafenib [6]; however, the improvement on the overall survival of patients with HCC is still limited. For example, sorafenib was shown to add around three months to the survival of patients with HCC in western countries, but only 2.3 months in patients in the Asian-Pacific region [10,11]. Identification of novel therapeutic targets of HCC for next-generation drug discovery and development is necessary and pressing.

AMP-activated protein kinase (AMPK), a highly conserved heterotrimeric serine/threonine kinase, serves as a eukaryotic cellular energy sensor and plays a vital role in the coordination of cell growth and metabolism. Under physiological condition, AMPK is regarded as a highly sensitive safeguard that responds to the changes in ATP production. Even with modest decreases in ATP production, AMPK could promote catabolic pathways to generate more ATP, and suppress anabolic signaling [12]. In mammals, AMPK has been shown to play essential roles in metabolic regulation in specialized tissues including liver, muscle, and fat [13]. In some pathological circumstances such as ischemia, hypoxia, low glucose level, or heat shock, AMPK is activated by an increased ratio of cellular AMP/ATP or ADP/ATP, and coordinates a series of cellular process including autophagy, apoptosis, cell cycle, cell metabolism as well as protein synthesis [14]. Malfunction and dysregulation of AMPK have been observed even different types of diseases, such as obesity [15], diabetes [16], aging [17], hypertension [18], heart failure [19], hepatic diseases [20] and certain types of cancers [21,22]. In particular, a growing body of research findings suggested that AMPK dysfunction may mediate a series of essential processes during the carcinogenesis and HCC progression. In this review, we critically summarized the recent findings identifying the role of AMPK in HCC. We also envisioned HCC treatment targeting AMPK according to the current understanding of the action AMPK activators. We hope our summary and discussion would shed light on the potential of AMPK as a therapeutic target in HCC treatment.

## 2. Structure and the Activation of AMPK

AMPK is a kind of heterotrimeric protein composed of a catalytic α subunit and two regulatory β and γ subunits [23]. Each of the subunits has two or three isoforms (α1 and α2; β1 and β2; γ1, γ2 and γ3) that are encoded by different genes [24]. The α subunit has a serine/threonine kinase structural domain as the principal regulatory site of AMPK activity and contains a central threonine activating site modulated by the upstream kinases [25]. The β subunit is thought to be the scaffold for α and γ subunits [26]. There are three AMP-binding domains in the γ subunit, one links with nucleotide and the other two links with ATP or AMP [26]. Moreover, the non-exchangeable nucleotide-binding sites include four repeats of a sequence motif named Cystathionine Beta Synthase (CBS) repeat [27].

**Figure 1:**
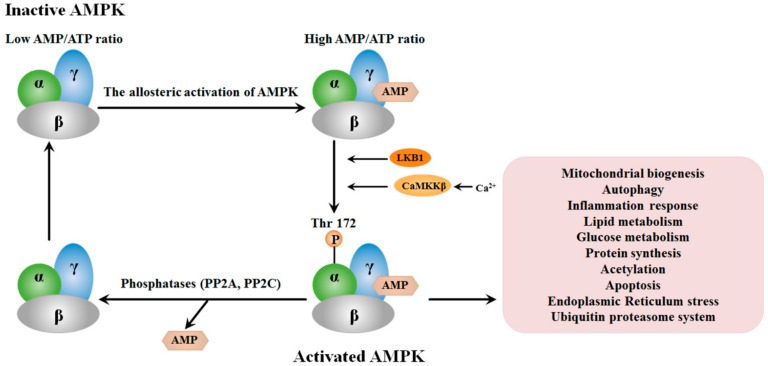
The structure and the activation of AMP-Activated Protein Kinase (AMPK). AMPK is composed of a catalytic α subunit and two regulatory β and γ subunits. The activation of AMPK relies on AMP-binding to the γ subunit and the phosphorylation of Thr172 on the α subunit regulated by upstream kinases like CaMKKβ and LKB1 and activated AMPK regulates its downstream signal pathways.

Three complementary mechanisms have been hypothesized in the activation of AMPK. First, the binding of AMP to the γ subunit leads to the allosteric activation of AMPK, and the increasing rate of the intracellular AMP:ATP motivates the substitution of ATP with AMP at the two exchangeable sites in the γ subunit. Second, the α subunit is phosphorylated in residue Thr172 by upstream kinases like Ca^2+^/calmodulin-dependent protein kinase β (CaMKKβ) and LKB1 [28,29]. Last but not least, the protein phosphatases (PP2A, PP2C) lead to the dephosphorylation of Thr172, which can be inhibited by AMP binding to AMPK [30]. Hence, the significant AMPK determinants activity are conformational switch induced by AMP and the α subunit phosphorylation on Thr172 (Figure 1). A series of reviews have reported the downstream signal pathways involving AMPK activity such as mitochondrial biogenesis, autophagy and inflammation response, as well summarized in other reviews [25,31,32]. Some downstream signal pathways of AMPK are shown in Figure 1.

## 3. Dysregulation of AMPK in HCC

It has always been reported that AMPK was aberrantly regulated during the carcinogenesis and progression of HCC, which was correlated with the aggressive clinicopathologic features and poor prognosis [14]. Cytokines in the tumor microenvironment may regulate AMPK expression and activity in HCC. CXCL17, a novel chemokine consisted of 119 amino acids, was found to suppress AMPK activity [33]. Yang et al. [34] analyzed the phosphorylation status of AMPK at Thr172 in the liver tissue of patients with cirrhosis. With 87-month follow-up, it was found that patients with low AMPK phosphorylation had a significantly higher incidence of HCC than patients with high AMPK phosphorylation levels (3.1/9.6/13.8/30.6% vs. 0/0.3/0.3/8% at 1/3/5/10 years after Hassab procedure; *p* < 0.001). Patients with p-AMPK low expression were estimated with a significantly higher risk of HCC occurrence in univariable analysis (Hazard ratio (HR), 6.25; 95% Confidence interval (CI): 3.36–11.60; *p* < 0.001) and multivariable analysis (HR, 6.0; 95% CI: 3.24–11.10; *p* < 0.001) [34]. In a cohort of 273 HCC patients including 253 with HBV history, low level of AMPK phosphorylation was found in 61.8% (81/131) of the patients. Low p-AMPK expression in HCC was correlated with high-serum α-fetoprotein (AFP) level, incomplete tumor encapsulation, late tumor–node–metastasis (TNM) stage, portal venous invasion, and distant metastasis [14]. In HCC patient receiving transcatheter arterial chemoembolization (TACE), the higher phosphorylation level of AMPK at Thr172 predicted improved disease-free and overall survival in a cohort of 378 Chinese HCC patients, while lower Thr172 phosphorylation indicated the presence of tumor-initiating cells in the liver [35]. Cai et al. analyzed the aberrantly methylated-differentially expressed genes in the Gene ominous database (https://www.ncbi.nlm.nih.gov/gds) of human HCC and found that genes enriched in AMPK pathways were significantly hypermethylated in HCC [36]. Divergent processes have been reported to attribute to AMPK down-regulation in HCC. Ketone catabolism in HCC cells was critical for the repression of AMPK activity under nutrient deprivation conditions [37]. The significant correlation between high SIRT1 activation and Thr172 phosphorylation of AMPK was found in HCC tissue harboring mutated p53 (*p* = 0.003, *n* = 57). Moreover, inactivation of SIRT1 was showed to inhibit AMPK pathway in HCC cells [38]. Oncogenic Ser/Thr protein phosphatase 5 (PP5) was also responsible for the decreased phosphorylated AMPK during hepatocarcinogenesis [39]. PP5 Inhibition reactivated AMPK signaling and suppressed HCC growth [40]. Deregulation of AMPK pathways in HCC may therefore play important role in cancer cell proliferation, survival, migration, invasion and metabolism (as summarized in Table S1).

## 4. The Regulation of AMPK on HCC

### 4.1. Regulation on Cell Proliferation

In HCC tissues, AMPK activity was negatively correlated with the tumor proliferation marker Ki67 [41], suggesting a role of AMPK in regulating HCC cell proliferation. Diverse mechanisms and signaling transductions have been investigated. Cell proliferation of mammalian HCC is commanded by the cell cycle progression, which is strictly regulated by the balance between cyclin-dependent kinases (CDKs), CDK inhibitors (CDKIs) and other growth suppressor proteins (GSPs) like p53 [42]. AMPK activation was found to induce G1/S phase cell cycle arrest of HCC cells, which was related to the increased expression of p27 and phosphorylation of Rb in HCC cells [43]. Another study on different HCC cell lines showed consistent G1/S arrest upon AMPK activation; however, this action of AMPK was most probably related to the up-regulation of p21 but not p27 [44]. Mechanistically, activation of AMPK triggered the phosphorylation of its downstream acetyl-CoA carboxylase carboxylase (ACC) to inhibit cell cycle regulators cyclin D1, CDK4 and CDK6 expression [45,46].

Other pathways are also involved in the regulation of AMPK on HCC cell proliferation. AMPK activation suppressed the transcription factor Sp1, which was responsible for the expression of DNMT1. DNMT1 silencing resulted in the reactivation of tumor suppressor genes and inhibited tumor growth [47,48,49]. β-catenin is a main oncogenic driver in HCC, which can regulate various of genes participant in cell development, growth, differentiation, and metastasis. The interaction between β-catenin and AMPK can regulate the proliferation and survival of HCC cells with selenium treatment [50,51]. AMPK activation blunted the protein translation-related mTOR signaling [52]. Other proliferation-related signaling that could be regulated by AMPK in HCC including ACC, p53 [53] and nuclear factor kappa-B (NF-κB) signaling [14].

YAP/TAZ is found as an attractive therapeutic target in HCC. The YAP/TAZ signaling modulator, NUAK2, also named as sucrose nonfermenting (SNF1)-like kinase (SNARK), is one of the AMPK-related kinases [54]. NUAK2 was identified as a key mediator of YAP-driven tumorigenesis and hepatomegaly in liver cancer models, whose pharmacological inactivation inhibited YAP-dependent liver overgrowth and cancer cell proliferation [55]. By promoting TGF-β signaling pathway, SNARK was reported to be a profibrogenic factor in HCC cells [56]. An anti-alcoholism drug disulfiram inhibited SNARK-promoted TGF-β signaling and exhibited anti-HCC effects [57].

### 4.2. Regulation on Cell Death

Hepatoma cells had LKB1 defects that blunted the AMPK signaling, and therefore were resistant to apoptosis induced by adenosine 3′,5′-cyclic monophosphate activation of protein kinase A and calcium/calmodulin-dependent protein kinase 2 [58]. This could be further proven with the evidence of inhibition of LKB1-downstream mTOR by AMPK activation [59], which initiated proliferator-activated receptor gamma coactivator 1-alpha (PGC-1α)-related HCC cell apoptosis [60]. Another study suggested that peroxisome proliferator-activated receptor γ (PPARγ) signaling could be activated by AMPK signaling, which was attributed to the increasing cell apoptosis [61]. Activation of AMPK in HCC was found to be associated with mitochondrial dysfunction and subsequent cell apoptosis [45,62]. This could be related to the endoplasmic reticulum stress and loss of mitochondrial membrane potential [63], which in turn activated the caspases cascades [64]. AMPK activation in HCC cells could also inactivate SIRT1, the p53 deacetylase to promote p53 acetylation and activation. [53,65]. In contrast, it was found that AMPK activation in HBV-related HCC cells could induce MnSOD expression, the enzyme that relieves intracellular redox stress and alleviates HCC cell apoptosis [66].

It was also suggested that AMPK activation in HCC resulted in transcription factor CCAAT/enhancer-binding protein delta (CEBPD) activation, which contributed to LC3B expression and induced autophagic cell death [67]. Another study suggested that the AMPK-induced autophagic cell death in HCC was dependent on the mTOR inhibition [68]. Interestingly, AMPK-induced autophagy in HCC was found to be linked with apoptotic cell death. Autophagy seemed to be necessary for the apoptosis initiation in HCC cells upon AMPK activation [69]. A further mechanistic study showed that the AMPK-induced autophagy was responsible for the lysosomal degradation of XIAP, the endogenous inhibitor of apoptosis, and promoted apoptotic cell death [70]. These findings suggested that both apoptosis and autophagy cooperatively contributed to the cell death of HCC upon AMPK activation.

### 4.3. Regulation on Cell Invasion and Metastasis

The reduced level of AMPK in HCC cells resulted in a new energy stress-inducible lncRNA MITA1, which in turn promoted Slug expression and epithelial-mesenchymal transition, the critical step of cancer metastasis [71]. AMPK suppression led to mTOR activation, which subsequently induced S6K/S6 signaling to promote cell motility [72]. Activation of AMPK was found to mediate the inhibition of HCC cell motility and invasiveness [73], as well as its adhesion to blood vessels [74]. AMPK activation suppressed Sp1 transcription factor, which was responsible for the invasion-related gene DNMT1 expression [47]. AMPK was also found to suppress JNK phosphorylation and Nanog expression induced by basic fibroblast growth factor (bFGF) in metastatic HCC cells [75].

### 4.4. Regulation of Cancer Metabolism

AMPK as a cell-intrinsic metabolic sensor can coordinate various metabolic process during the hepatocarcinogenesis and progression of HCC. MATα1:MATα2 switch in the liver human repressed AMPK signaling to deregulate the methionine metabolism [76]. Further study showed that AMPK was essential to suppress the NF-κB activation and signal transducer and activator of transcription 3 (STAT3) in response to metabolic stress during hepatocarcinogenesis, and prevented the dysregulation of glycogen storage and formation of the hepatic tumor [77]. HCC cells could also re-activate ketone catabolism, which repressed AMPK activity and protected tumor cells from excessive autophagic cell death [37]. In addition to its well-known effect in regulating glucose metabolism, AMPK was reported to participate in the regulation on the metabolism of cellular fatty acid and protein. Mice with functional loss of mutation on AMPK showed accelerated de novo lipogenesis and formation of the liver lesion [78]. AMPK activation mediated the inhibition of nascent protein synthesis and retarded HCC proliferation [79]. However, in HBV-associated carcinogenesis of HCC, AMPK activation was proposed to have a tumor-promoting role. HBX protein induced a hyperactivation of AMPK and its downstream signaling, which re-directed the metabolic pathway in hepatocytes to facilitate persistent HBV replication. AMPK inhibition showed to reduce HBV DNA replication [80]. Further study showed that HBX-activated AMPK could initiate calcium/CaMKK-dependent pathway-dependent fatty acid oxidation to maintain the cellular NADPH and ATP level for HBV replication [80].

## 5. AMPK Activators in HCC Treatment

**Figure 2:**
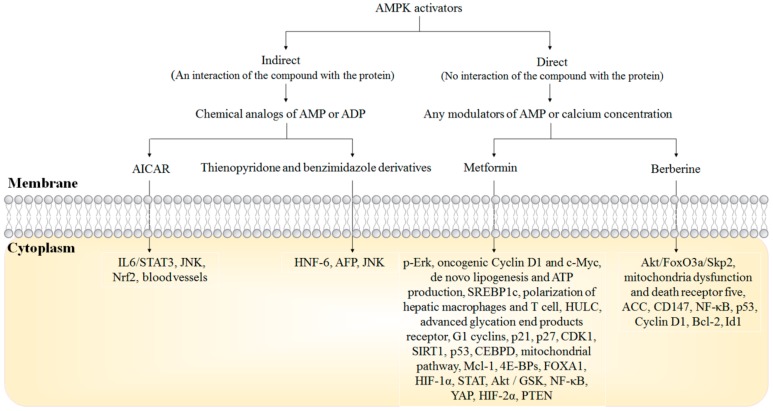
The schematic of AMPK activators regulate Hepatocellular Carcinoma (HCC) development.

Indirect and direct activators of AMPK have been discovered and developed. Direct activators of AMPK require an interaction between the compounds and the protein, leading to the conformational change of AMPK. Most of the direct AMPK activators are the chemical analogs of AMP or ADP. Indirect activators of AMPK can be practically any modulators of AMP or calcium concentration, which allows the interaction between the compound and AMPK protein not necessary [81]. Several AMPK activators have been extensively studied for their anti-HCC activities, including indirect activators metformin and berberine, and direct activators 5-aminoimidazole-4-carboxamide riboside (AICAR), thienopyridone (A-769662) and benzimidazole (compound 911) derivatives (Figure 2).

### 5.1. Metformin

In AKT/c-Met-triggered HCC mice models, metformin obstructed the malignant transformation of hepatocytes and consequently delayed HCC initiation [82]. This was related to the reduced p-Erk and expression of oncogenic Cyclin D1 and c-Myc, as well as the suppression of fatty acid synthase-induced de novo lipogenesis and ATP production.

In vitro study confirmed that the action of metformin was AMPK- and SREBP1c-dependent [82,83]. This effect of metformin was similarly observed in high fat and fructose diet and diethylnitrosamine-induced hepatocarcinogenesis [84,85]. In a zebrafish model of NAFLD/NASH, metformin treatment was associated with reduced hepatocarcinogenesis, which was attributed to its regulation on the polarization of hepatic macrophages as well as T cell population [86]. In HBV-associated hepatocarcinogenesis, metformin was able to repress the HBX-induced HULC expression, resulting in reduced HCC occurrence and progression [87]. Mechanistically, early intervention of metformin during hepatocarcinogenesis could repress the receptor for advanced glycation end products and inhibit the activation of hepatic progenitor cells [88].

Myoshi et al. found that metformin could induce G0/G1 cell cycle arrest in HCC cells, which was related to the inhibition of G1 cyclins [89], as well as the up-regulation of p21 and p27 [90]. Suppression of HCC cell proliferation by metformin was also attributed to the up-regulation of miR-378, which in turn repressed CDK1 expression and induced G2/M cell cycle arrest [91]. Zhou et al. suggested that metformin-activated AMPK reduced SIRT1 activity, leading to cell senescence instead of apoptosis in HCC xenografts, and resulting in tumor inhibition [92]. The metformin-induced senescence in HCC cells was p53-dependent [53]. Tsai et al. found that metformin activated transcription factor CEBPD via AMPK, which initiated transcription of LC3B and Atg3 and induced autophagy-dependent apoptosis in HCC cells [67]. Apoptosis induced by metformin was dependent to the mitochondrial pathway [93]. Bhat et al. suggested that metformin-induced HCC cell apoptosis and growth inhibition be related to translation inhibition of Mcl-1 oncoprotein and activation of 4E-BPs [94]. Sun et al. suggested that metformin-induced AMPK could stabilize p53, which induced miR-23a to suppress FOXA1 pathway and initiated apoptosis [95]. Metformin-induced AMPK could also decrease Livin expression in HCC and promote cell apoptosis [96]. Metformin also possessed an AMPK-independent anti-proliferative activity against HCC, which could be related to the reduction of oxygen consumption and increased intracellular oxygen tension via down-regulation of HIF-1α [97]. Besides, inhibition of in vivo tumor of HCC by metformin could be associated with the decreased Th1- and Th17-derived IL-22 production through AMPK-dependent STATs down-regulation [98].

Ferritti et al. suggested that metformin suppress the migration and invasion of hepatocellular carcinoma cells dependent on AMPK activation [99]. This effect of metformin was related to its blockade on bFGF-induced Akt/GSK activation, and subsequent Twist1 stabilization [100]. Meanwhile, metformin repressed HCC metastasis through inhibiting tumor angiogenesis. This was related to the inhibition of hepatic stellate cells interaction with vascular endothelial cells in an AMPK-dependent manner [101].

Studies also revealed the potential of metformin as adjuvant therapy. Metformin can suppress the expression of several multidrug resistance proteins, probably through inhibiting NF-κB signaling [102]. Metformin reduced YAP expression and activity and sensitized HCC cells to 5-Fu treatment [103]. Metformin may also potentiate arsenic trioxide toxicity through down-regulation of Bcl-2 in HCC cells [104]. Co-treatment of metformin with regorafenib or sorafenib showed high potency in inhibiting HCC cells, which was associated with the down-regulation of HIF-2α and subsequent up-regulation of TIF30 [105,106,107]. Hsieh et al. showed that AMPK/NF-κB -dependent inhibition of uPA and MMP9 was also involved [108]. Zhang et al. suggested that metformin could block the proliferation and invasion of HCC cells after insufficient radiofrequency ablation. This effect of metformin could be associated with the suppression of Akt survival signaling through AMPK/PTEN [109].

### 5.2. Berberine

Several lines of evidence have suggested the anti-HCC potential of berberine with multiple mechanisms [110,111,112]. Berberine can induce G0/G1 cell cycle arrest in HCC cells, which was associated with its inhibition on Akt/FoxO3a/Skp2 axis to promote the expression of endogenous CDKi p21 and p27 [113]. The induction of cell apoptosis of HCC by berberine could be AMPK-dependent, which involved mitochondria dysfunction and death receptor five as downstream pathways [64,114]. Induction of autophagic and apoptotic cell death of HCC by berberine was AMPK-dependent, in which ACC acted as the downstream regulator [115]. Hou et al. found that CD147 inhibition was also involved in autophagic and apoptotic cell death induced by berberine [116]. Li et al. showed that arachidonic acid (AA) metabolic pathway was also the target of berberine in inducing HCC cell apoptosis [117]. Another study suggested that AA-associated NF-κB was involved [112]. Berberine could induce expression of miR-21a-3p, which targeted on methionine adenosyltransferase (MAT) to induce apoptosis of HCC cells [118]. Wang et al. showed that another p53-inducible miRNA, miRNA-23a was also involved in berberine’s action in inhibiting HCC by targeting Nek6 [111]. Another miRNA, miR-22, was found to be up-regulated by berberine in HCC, which in turn suppressed cell cycle progression and tumor survival by targeting Cyclin D1 and Bcl-2 [119]. Tsang et al. suggested that Id1 was the principal target of HCC in vivo, in which berberine suppressed Id1 expression to block tumor growth and lung metastasis [120]. The anti-invasive effect of berberine in vitro could be primarily dependent to PAI-1 up-regulation and uPA down-regulation in HCC [121]. Jie et al. showed that berberine treatment could inhibit the VEGF expression in HCC cells, which contributed to the tumor angiogenesis [122]. Furthermore, berberine was found to sensitize HCC cells to chemotherapeutic treatment. Guo et al. suggested that inhibition of CD147 by berberine contributed to the rapamycin sensitivity in HCC cells [123]. Huang et al. showed that berberine could improve sorafenib toxicity to HCC cells by down-regulating the Bcl-2 protein expression [124].

### 5.3. AICAR

AICAR, a kind of cell-permeable nucleoside, is the antepenultimate metabolic intermediate of the de novo purine synthesis pathway, which was used as the AMPK activator [125]. AICAR was found to repress tumorigenesis in the liver through suppressing IL6/STAT3 activation [126]. In the established HCC, AICAR treatment resulted in HepG2 cell cycle arrest at G1/G1 phase. AICAR inhibited the stem-like cell marker NANOG expression in HepG2 cells via suppressing JNK activity, which suggested the potential of AICAR in inhibiting tumor-initiating cells in HCC [75]. AICAR induced the Nrf2 expression and modulated the cellular redox homeostasis in HCC cells. This effect of AICAR was AMPK-dependent [125]. AICAR activation could significantly inhibit the adhesion of HCC cells onto vascular endothelial cells HUVECs, indicating the ability of AICAR in inhibiting HCC cell migration through blood vessels [74].

### 5.4. Thienopyridone and Benzimidazole Derivatives

Conventional cytotoxic drugs for HCC like fluorouracil show almost no improvement in survival with severe adverse effects. Sorafenib was successfully found to extend survival of patients with advanced HCC, suggesting a promising strategy of small-molecule targeted chemotherapy [127]. A series of thienopyridone derivatives was synthesized and showed potent activity in killing HCC. The IC50 of these novel compounds was as low as 10 nM [127]. Zhou et al. synthesized a novel thienopyridine derivative compound named TP58. It was found that TP58 could induce G1/S cell cycle arrest in HCC cells with 16 genes being regulated. Further analysis showed that TP58 could regulate some liver-specific transcriptional factors and HCC-specific markers such as HNF-6 and AFP [128]. El-Miligy and colleague synthesized several benzimidazole derivatives, which exhibited dual inhibition on HCV replication and HCC growth, suggesting the prophylactic and curative potential of these compounds on liver cancer [129]. Dai et al. suggested a novel benzimidazole derivative methyl 2-(5-fluoro-2-hydroxyphenyl)-1H-benzo[d]imidazole-5-carboxylate (MBIC) could induce HCC cells apoptosis without affecting the normal liver cells. MBIC could initiate intracellular reactive species oxygen and activated JNK pathway, which contributed as the main mechanism of MBIC-induced apoptosis. In animals, 25 mg/kg MBIC significantly blocked HCC growth [130].

## 6. Clinical Perspective of AMPK Activation in HCC Treatment

Given the accumulating pre-clinical evidence showing that AMPK activation could suppress HCC, clinical studies on the efficacy and effectiveness of AMPK activators alone or in combination in treating HCC have been launched. Metformin, as the commonly used anti-diabetics, was generally selected as the pioneer AMPK activator since its well-documented pharmacokinetic and safety profiles. In a recent retrospective study including 5093 patients with HCC, those of whom received metformin treatment showed an improved hepatic function (Child-Pugh-Score A: 69.2% vs. 47.4%; *p* < 0.001) and underwent more often tumor resection (22.1% vs. 16.5%; *p* < 0.05) [131]. A nationwide population study in Korea showed that the use of metformin was associated with improvement of HCC-specific mortality and reduced occurrence of retreatment events in HCC patients with curative resection [132]. However, the improvement of survival by metformin could not be observed in another study in the United State [133]. metformin showed no beneficial effect in prolonging survival of patients with HCC receiving sorafenib treatment [134]. This might be associated with the increased SIRT3 expression in patients receiving metformin [135]. Another clinical study about patients with advanced HCC revealed worse outcomes with concomitant sorafenib and metformin intervention compared with sorafenib alone, which suggested that the co-treatment of sorafenib and metformin was not helpful in spite of harmless effects [134]. There are several registered on-going clinical trials on the potential use of metformin on HCC.

## 7. Conclusions

An increasing number of studies have suggested that the intracellular energy sensor signaling AMPK plays an important role in the regulation of human HCC. AMPK signaling was found to be down-regulated in HCC, at kinase activity and expression levels. Down-regulation of AMPK in tumor tissues was associated with the loss of control in tumorigenesis, cell cycle progression, proliferation and survival, invasion and metastasis and cancer metabolism and drug resistance. AMPK activation led to HCC suppression in cell and animal studies. Several AMPK activators, such as metformin, berberine, AICAR, and some thienopyridone/benzimidazole derivatives, were found to be effective in suppressing HCC with their function dependent or independent to AMPK activities, and a few clinical evidence have been gained for the potential repurposing of metformin in HCC treatment. Further translational and clinical investigations are urgently necessary to shed light on the potential of AMPK activators in HCC therapy.

## Supplementary Materials

The following are available online at https://www.mdpi.com/2072-6694/11/5/647/s1, Table S1: Regulation of AMPK on HCC.

## Abbreviations

AA	Arachidonic acid
ACC	Acetyl-CoA carboxylase carboxylase
AFP	α-fetoprotein
AICAR	5-aminoimidazole-4-carboxamide riboside
bFGF	Basic fibroblast growth factor
CaMKK	Ca^2+^/calmodulin-dependent protein kinase
CDKs	Cyclin-dependent kinases
CDKIs	CDK inhibitors
CEBPD	CCAAT/enhancer-binding protein delta
GSPs	Growth suppressor proteins
HCC	Hepatocellular carcinoma
MAT	Methionine adenosyltransferase
MBIC	Methyl 2-(5-fluoro-2-hydroxyphenyl)-1*H*-benzo[d]imidazole-5-carboxylate
NAFLD	Nonalcoholic fatty liver disease
NF-κB	Nuclear factor kappa-B
PP5	Protein phosphatase 5
PPARγ	Peroxisome proliferator-activated receptor γ
PGC-1α	Proliferator-activated receptor gamma coactivator 1-alpha
STAT3	Signal transducer and activator of transcription 3
TACE	Transcatheter arterial chemoembolization
TNM	Tumor–node–metastasis
