# Supplementary material for: The Role of AMP-Activated Protein Kinase as a Potential Target of Treatment of Hepatocellular Carcinoma
Source: Cancers (Basel). 2019 May 10;11(5):647. doi: 10.3390/cancers11050647 (PMC6562911; doi:10.3390/cancers11050647)
Supplement: Supplementary file 1 [file cancers-11-00647-s001.pdf]

Supplemental Materials: The Role of AMP-Activated Protein Kinase as a Potential Target of Treatment of Hepatocellular Carcinoma

Xue Jiang, Hor-Yue Tan, Shanshan Teng, Yau-Tuen Chan, Di Wang and Ning Wang

Table S1. Regulation of AMPK on HCC.

Actions	Models	Regulation/pathway	Effects	Reference.
Dysregulation of AMPK in HCC	In clinic, cirrhotic liver tissues; In vivo, C57BL/6 mice	Autophagy-related	Negative correlation between AMPK activation and HCC occurrence	[1] (2018)
	In clinic, normal liver tissues obtained from the distal normal liver tissue of patients with liver hemangioma; Formalin-fixed and paraffin-embedded HCC tissues from 273 consecutive patients and fresh-frozen HCC tissues from 19 patients who underwent radical resection	NF-κB and STAT3 signaling	Activation of AMPK inhibited HCC cells growth	[2] (2013)
	In clinic, 378 HCC patients	High percentage of OV6+ tumor-initiating cells (T-ICs) in HCC specimens	High pAMPKα (Thr172) level may serve as a positive predictor of survival in HCC patients undergoing TACE	[3] (2016)
	In vitro, human HepG2, Hep3B, PLC, HEK293 and HEK293T cells; In vivo, nude mice	Ketolysis and autophagy	Nutrition-deprived HCC cells employ ketone bodies for energy supply and cancer progression	[4] (2016)
	In clinic, patients with HCC; In vitro, PLC/PRF/5, HepG2 and Hep3B cells; In vivo, NOC male mice	AMPK-mTOR pathway	Metformin could be a therapeutic drug for HCC in patients with mutated p53, inactivated SIRT1, and AMPK expression	[5] (2015)
	In vitro, Hep3B and PLC/PRF/5(PLC5) cell lines; In vivo, 5-week-old male NCr athymic nude mice	Oncogenic PP5 inhibition	AMPK activation by inhibiting PP5 for anti-HCC therapy	[6] (2017)
	In clinic, 153 primary HCC tumor tissues and a paired normal tissue; In vivo, Hep3B, PLC5, and Huh7 cells	PP5/AMPK axis independent of CDK4/6	Palbociclib induced autophagy and apoptosis in HCC cells	[7] (2017)
	In vitro, HepG2 and SK-Hep1 cells	SIRT1/AMPK	Gallotannin-induced senescence and impaired autophagy leading to cell death in HCC cells	[8] (2017)
	In vitro, the human HCC cell line HCCLM3, HepG2, SK-Hep1, Huh7, and the normal liver cell line L02	PKM2-AMPK-PGC1α signaling	Shikonin exerts antitumor activity by causing mitochondrial dysfunction in HCC	[9] (2018)
	In vitro, HCC cells	AMPK-mTOR-S6 K1 signaling	ATIC suppresses AMPK activation and supports growth and motility activity of HCC cells	[10] (2017)
Regulation on cell proliferation;	In clinic, the human tissue samples; In vitro, the human hepatoma cell lines, PLC/PRF/5 and HepG2; In vivo, BALB/c-nu mice	Metabolism and the cell cycle	AMPK activity inhibits the proliferation of HCC	[11] (2014)

Regulation on cell death	In clinic, liver tumor specimens and adjacent non-tumorous specimens; In vitro, the human hepatoma cell lines HepG2, SK-Hep1, and PLC/PRF/5; In vivo, BALB/c nude mice	AMPK/p27Kip1 axis, CDK/Rb/E2F pathway	PCK1 negatively regulates cell cycle progression and hepatoma cell proliferation	[12] (2019)
	In vitro, HCC cell lines, HepG2 and Hep3B; In vivo, male BALB/c nude mice	Upregulated p21 and p27 by regulating AMPK and STAT3-Skp2 axis	Simvastatin induces G0/G1 arrest	[13] (2017)
	In vitro, the HepG2 cell line	AMPK and the downstream acetyl-CoA carboxylase carboxylase	Fatsioside A induced apoptotic death of HepG2 HCC cells	[14] (2015)
	In vitro, HLF cells	ACC and cyclin D1, CDK4 and CDK6 expression	Fucoidan inhibits proliferation through AMPK-associated suppression of fatty acid synthesis and G1/S transition in HLF cells	[15] (2015)
	In vitro, the cancer cell lines including HepG2, PLC/PRF/5 and Hep3B	mTOR translational pathway	G1 arrest of the cell-cycle and subsequent cell death	[16] (2010)
	In vitro, human liver cancer cell lines HepG2 and Bel-7402	A p53-dependent manner	Exposure of hepatoma cells to low doses of metformin results in the induction of senescence instead of apoptosis	[17] (2013)
	In vitro, HCC cells	AMPKα-mediated inhibition of Sp1 and DNMT1	Ursolic acid inhibits growth of HCC	[18] (2014)
	In vitro, the Hep3B HCC cell line	GSK3β-independent AMPK/β-catenin pathway	Selenium down-regulates the β-catenin survival pathway through activation of AMPK in hepatocellular carcinoma cells and xenograft tumor models	[19] (2016)
	In vitro, human Huh7 and Bel7402 cells; In vivo, male BALB/c nude mice	mitochondria dysfunction and endoplasmic reticulum stress	Physcion had pro-apoptotic role on HCC cells	[20] (2018)
	In vitro, the human HCC cell lines (HepG2, SMMC-7721 and Bel-7402) and the normal liver cell line (HL-7702)	AMPK-mediated caspase-dependent mitochondrial pathway cell apoptosis	AMPK may be involved in the antitumor effect of berberine	[21] (2013)
	In vitro, human SMMC7721 and Bel7402 cell lines; In vivo, male athymic BALB/c nu/nu mice	Activation of PPARγ	PPARγ activation by hispidulin effectively suppressed HCC cell growth and metastasis	[22] (2018)
	In vitro, the human hepatoma cell lines, HepG2 and PLC/PRF/5	Destabilising p53 in a SIRT1-dependent manner	Inactivation of AMPK promotes hepatocarcinogenesis	[23] (2012)
	In vitro, the human hepatoma cell line HepG2	AMPK/MnSOD signaling	HBV suppresses mitochondrial superoxide level and exerts an anti-apoptotic effect in HBV-infected HepG2 cells	[24] (2016)
	In vitro, human HCC cell lines Huh7, HepG2 and Hep3B; In vivo, male NOD/SCID mice	The tumor suppressor CEBPD expression	The combinatorial treatment of metformin and rapamycin can enhance autophagic cell death of HCC	[25] (2017)

	In vitro, human HCC cell lines used, HepG2, Bel-7402, Huh-7, SMMC-7721 and SMMC-7402	GRP78–AMPK–mTOR signaling	Endoplasmic reticulum stress and autophagy were involved in cell death evoked by meloxicam in HCC cells	[26] (2015)
	In vitro, the human normal liver cell line (L02 cells) and the HCC cell lines (HepG2 and SMMC-7721 cells)	Autophagy-induced apoptosis via AMPK signaling	SCD1 regulates autophagy via AMPK signaling	[27] (2014)
	In vitro, the human HCC cell lines HepG2, PLC/PRF/5 and Hep3B; MHCC97L cell line; the human normal hepatic cell line L-02	AMPKα/mTOR-dependent autophagy and the triggered XIAP heading lysosomal degradation pathway	Timosaponin AIII induces HCC cell apoptosis through a p53-independent mechanism	[28] (2013)
Regulation on cell invasion and metastasis	In clinic, liver tumors and paired adjacent normal livers; In vitro, HepG2, A549, U87 and PC3 cell lines; Huh7, HCCLM3, SK-Hep1, SMMC-7721, LO2, HGC-27 and U251 cell lines; In vivo, female NOD/SCID mice	AMPK-MITA1-Slug axis	MITA1 is a crucial driver of HCC metastasis	[29] (2019)
	In clinic, HCC tissue specimens and adjacent normal tissues; In vitro, human normal hepatic cell LO-2 cells and HCC HepG2.2.15 cells	The AMPK signaling pathway by targeting CCNA1	MiR-1271 overexpression conferred protection against cell proliferation, migration, and invasion in HBV-associated HCC	[30] (2019)
	In vitro, the HCC SK-Hep1 cells, human umbilical vein endothelial cells (HUVECs)	The resistin effect on SK-Hep1 cell adhesion to HUVECs	The inhibitory effect of AMPK activation under the resistin stimulation	[31] (2014)
Regulation of cancer metabolism	In vivo, the rAAV-infused G6pc−/− mice	AMPK/sirtuin-1 and FGF21/β-klotho signalings combined with down-regulation of STAT3/NFκB-mediated inflammatory and tumorigenic signaling	The absence of hepatic tumors in AAV-NT mice	[32] (2017)
	In vitro, HepG2 (ATCC HB-8065) human liver cancer; In vivo, ACC KI mice, male Wistar rats	AMPK-mediated ACC phosphorylation	The importance of DNL and dysregulation of AMPK-mediated ACC phosphorylation in accelerating HCC	[33] (2019)
	In vitro, human HCC cell line HepG2 and MHCC97L; In vivo, female BALB/c nu/nu athymic nude mice	eEF2K/AMPK	Huanglian Jiedu Decoction mediates eEF2 inactivation in HCC cells	[34] (2015)
	In vitro, primary rat hepatocyte system	AMPK-mTORC1 axis	Inhibition of AMPK decreased HBV replication	[35] (2016)

References

1. Yang, X.; Liu, Y.; Li, M.; Wu, H.; Wang, Y.; You, Y.; Li, P.; Ding, X.; Liu, C.; Gong, J. Predictive and preventive significance of AMPK activation on hepatocarcinogenesis in patients with liver cirrhosis. *Cell Death Disease* **2018**, *9*, 264, doi:10.1038/s41419-018-0308-4.
2. Zheng, L.; Yang, W.; Wu, F.; Wang, C.; Yu, L.; Tang, L.; Qiu, B.; Li, Y.; Guo, L.; Wu, M.; et al. Prognostic significance of AMPK activation and therapeutic effects of metformin in hepatocellular carcinoma. *Clin. Cancer Res.* **2013**, *19*, 5372–5380, doi:10.1158/1078-0432.CCR-13-0203.
3. Zheng, L.Y.; Wu, L.; Lu, J.; Zou, D.J.; Huang, Q. Expression of Phosphorylated AMP-Activated Protein Kinase Predicts Response to Transarterial Chemoembolization in Postoperative Cases of Hepatocellular Carcinoma. *Medicine* **2016**, *95*, e2908, doi:10.1097/MD.0000000000002908.
4. Huang; Li, T.; Wang, L.; Zhang, L.; Yan, R.; Li, K.; Xing, S.; Wu, G.; Hu, L.; Jia, W.; et al. Hepatocellular carcinoma redirects to ketolysis for progression under nutrition deprivation stress. *Cell Res.* **2016**, *26*, 1112–1130, doi:10.1038/cr.2016.109.
5. Zhang, Z.Y.; Hong, D.; Nam, S.H.; Kim, J.M.; Paik, Y.H.; Joh, J.W.; Kwon, C.H.; Park, J.B.; Choi, G.S.; Jang, K.Y.; et al. SIRT1 regulates oncogenesis via a mutant p53-dependent pathway in hepatocellular carcinoma. *J. Hepatol.* **2015**, *62*, 121–130, doi:10.1016/j.jhep.2014.08.007.
6. Chen, Y.L.; Hung, M.H.; Chu, P.Y.; Chao, T.I.; Tsai, M.H.; Chen, L.J.; Hsiao, Y.J.; Shih, C.T.; Hsieh, F.S.; Chen, K.F. Protein phosphatase 5 promotes hepatocarcinogenesis through interaction with AMP-activated protein kinase. *Biochem. Pharmacol.* **2017**, *138*, 49–60, doi:10.1016/j.bcp.2017.05.010.
7. Hsieh, F.S.; Chen, Y.L.; Hung, M.H.; Chu, P.Y.; Tsai, M.H.; Chen, L.J.; Hsiao, Y.J.; Shih, C.T.; Chang, M.J.; Chao, T.I.; et al. Palbociclib induces activation of AMPK and inhibits hepatocellular carcinoma in a CDK4/6-independent manner. *Mol. Oncol.* **2017**, *11*, 1035–1049, doi:10.1002/1878-0261.12072.
8. Kwon, H.Y.; Kim, J.H.; Kim, B.; Srivastava, S.K.; Kim, S.H. Regulation of SIRT1/AMPK axis is critically involved in gallotannin-induced senescence and impaired autophagy leading to cell death in hepatocellular carcinoma cells. *Arch. Toxicol.* **2018**, *92*, 241–257, doi:10.1007/s00204-017-2021-y.
9. Liu, B.; Jin, J.; Zhang, Z.; Zuo, L.; Jiang, M.; Xie, C. Shikonin exerts antitumor activity by causing mitochondrial dysfunction in hepatocellular carcinoma through PKM2-AMPK-PGC1alpha signaling pathway. *Biochem. Cell Bio.* **2018**, 10.1139/bcb-2018-0310, doi:10.1139/bcb-2018-0310.
10. Li, M.; Jin, C.; Xu, M.; Zhou, L.; Li, D.; Yin, Y. Bifunctional enzyme ATIC promotes propagation of hepatocellular carcinoma by regulating AMPK-mTOR-S6 K1 signaling. *Cell Commun. Signal.* **2017**, *15*, 52, doi:10.1186/s12964-017-0208-8.
11. Cheng, J.; Huang, T.; Li, Y.; Guo, Y.; Zhu, Y.; Wang, Q.; Tan, X.; Chen, W.; Zhang, Y.; Cheng, W.; et al. AMP-activated protein kinase suppresses the in vitro and in vivo proliferation of hepatocellular carcinoma. *PloS ONE* **2014**, *9*, e93256, doi:10.1371/journal.pone.0093256.
12. Tuo, L.; Xiang, J.; Pan, X.; Hu, J.; Tang, H.; Liang, L.; Xia, J.; Hu, Y.; Zhang, W.; Huang, A.; et al. PCK1 negatively regulates cell cycle progression and hepatoma cell proliferation via the AMPK/p27(Kip1) axis. *J. Exp. Clin. Cancer Res.* **2019**, *38*, 50, doi:10.1186/s13046-019-1029-y.
13. Wang, S.T.; Ho, H.J.; Lin, J.T.; Shieh, J.J.; Wu, C.Y. Simvastatin-induced cell cycle arrest through inhibition of STAT3/SKP2 axis and activation of AMPK to promote p27 and p21 accumulation in hepatocellular carcinoma cells. *Cell Death Dis.* **2017**, *8*, e2626, doi:10.1038/cddis.2016.472.
14. Zheng, Y.S.; Zhang, J.Y.; Zhang, D.H. Fatsioside Ainduced apoptotic death of HepG2 cells requires activation of AMPactivated protein kinase. *Mol. Med. Rep.* **2015**, *12*, 5679–5684, doi:10.3892/mmr.2015.4194.
15. Kawaguchi, T.; Hayakawa, M.; Koga, H.; Torimura, T. Effects of fucoidan on proliferation, AMP-activated protein kinase, and downstream metabolism- and cell cycle-associated molecules in poorly differentiated human hepatoma HLF cells. *Int. J. Oncol.* **2015**, *46*, 2216–2222.
16. Chiang, P.C.; Lin, S.C.; Pan, S.L.; Kuo, C.H.; Tsai, I.L.; Kuo, M.T.; Wen, W.C.; Chen, P.; Guh, J.H. Antroquinonol displays anticancer potential against human hepatocellular carcinoma cells: a crucial role of AMPK and mTOR pathways. *Biochem. Pharmacol.* **2010**, *79*, 162–171, doi:10.1016/j.bcp.2009.08.022.
17. Yi, G.; He, Z.; Zhou, X.; Xian, L.; Yuan, T.; Jia, X.; Hong, J.; He, L.; Liu, J. Low concentration of metformin induces a p53-dependent senescence in hepatoma cells via activation of the AMPK pathway. *Int. J. Oncol.* **2013**, *43*, 1503–1510, doi:10.3892/ijo.2013.2077.

18. Yie, Y.; Zhao, S.; Tang, Q.; Zheng, F.; Wu, J.; Yang, L.; Deng, S.; Hann, S.S. Ursolic acid inhibited growth of hepatocellular carcinoma HepG2 cells through AMPKalpha-mediated reduction of DNA methyltransferase 1. *Mol. Cell. Biochem.* **2015**, *402*, 63–74, doi:10.1007/s11010-014-2314-x.
19. Park, S.Y.; Lee, Y.K.; Kim, H.J.; Park, O.J.; Kim, Y.M. AMPK interacts with beta-catenin in the regulation of hepatocellular carcinoma cell proliferation and survival with selenium treatment. *Oncol. Rep.* **2016**, *35*, 1566–1572, doi:10.3892/or.2015.4519.
20. Pan, X.P.; Wang, C.; Li, Y.; Huang, L.H. Physcion induces apoptosis through triggering endoplasmic reticulum stress in hepatocellular carcinoma. *Biomed. Pharmacother.* **2018**, *99*, 894–903, doi:10.1016/j.biopha.2018.01.148.
21. Yang, X.; Huang, N. Berberine induces selective apoptosis through the AMPKmediated mitochondrial/caspase pathway in hepatocellular carcinoma. *Mol. Med. Rep.* **2013**, *8*, 505–510, doi:10.3892/mmr.2013.1506.
22. Han, M.; Gao, H.; Ju, P.; Gao, M.Q.; Yuan, Y.P.; Chen, X.H.; Liu, K.L.; Han, Y.T.; Han, Z.W. Hispidulin inhibits hepatocellular carcinoma growth and metastasis through AMPK and ERK signaling mediated activation of PPARgamma. *Biomed. Pharmacother.* **2018**, *103*, 272–283, doi:10.1016/j.biopha.2018.04.014.
23. Lee, C.W.; Wong, L.L.; Tse, E.Y.; Liu, H.F.; Leong, V.Y.; Lee, J.M.; Hardie, D.G.; Ng, I.O.; Ching, Y.P. AMPK promotes p53 acetylation via phosphorylation and inactivation of SIRT1 in liver cancer cells. *Cancer Res.* **2012**, *72*, 4394–4404, doi:10.1158/0008-5472.CAN-12-0429.
24. Li, L.; Hong, H.H.; Chen, S.P.; Ma, C.Q.; Liu, H.Y.; Yao, Y.C. Activation of AMPK/MnSOD signaling mediates anti-apoptotic effect of hepatitis B virus in hepatoma cells. *World J. Gastroenterol.* **2016**, *22*, 4345–4353, doi:10.3748/wjg.v22.i17.4345.
25. Tsai, H.H.; Lai, H.Y.; Chen, Y.C.; Li, C.F.; Huang, H.S.; Liu, H.S.; Tsai, Y.S.; Wang, J.M. Metformin promotes apoptosis in hepatocellular carcinoma through the CEBPD-induced autophagy pathway. *Oncotarget* **2017**, *8*, 13832–13845, doi:10.18632/oncotarget.14640.
26. Zhong, J.; Dong, X.; Xiu, P.; Wang, F.; Liu, J.; Wei, H.; Xu, Z.; Liu, F.; Li, T.; Li, J. Blocking autophagy enhances meloxicam lethality to hepatocellular carcinoma by promotion of endoplasmic reticulum stress. *Cell Proliferation* **2015**, *48*, 691–704, doi:10.1111/cpr.12221.
27. Huang, G.M.; Jiang, Q.H.; Cai, C.; Qu, M.; Shen, W. SCD1 negatively regulates autophagy-induced cell death in human hepatocellular carcinoma through inactivation of the AMPK signaling pathway. *Cancer Letters* **2015**, *358*, 180–190, doi:10.1016/j.canlet.2014.12.036.
28. Wang, N.; Feng, Y.; Zhu, M.; Siu, F.M.; Ng, K.M.; Che, C.M. A novel mechanism of XIAP degradation induced by timosaponin AIII in hepatocellular carcinoma. *Biochim. Biophys. Acta.* **2013**, *1833*, 2890–2899, doi:10.1016/j.bbamcr.2013.07.018.
29. Ma, M.; Xu, H.; Liu, G.; Wu, J.; Li, C.; Wang, X.; Zhang, S.; Xu, H.; Ju, S.; Cheng, W.; et al. MITA1, a novel energy stress-inducible lncRNA, promotes hepatocellular carcinoma metastasis. *Hepatology* **2019**, 10.1002/hep.30602, doi:10.1002/hep.30602.
30. Chen, Y.; Zhao, Z.X.; Huang, F.; Yuan, X.W.; Deng, L.; Tang, D. MicroRNA-1271 functions as a potential tumor suppressor in hepatitis B virus-associated hepatocellular carcinoma through the AMPK signaling pathway by binding to CCNA1. *J. Cellular Physiol.* **2019**, *234*, 3555–3569, doi:10.1002/jcp.26955.
31. Yang, C.C.; Chang, S.F.; Chao, J.K.; Lai, Y.L.; Chang, W.E.; Hsu, W.H.; Kuo, W.H. Activation of AMP-activated protein kinase attenuates hepatocellular carcinoma cell adhesion stimulated by adipokine resistin. *BMC Cancer* **2014**, *14*, 112, doi:10.1186/1471-2407-14-112.
32. Kim, G.Y.; Kwon, J.H.; Cho, J.H.; Zhang, L.; Mansfield, B.C.; Chou, J.Y. Downregulation of pathways implicated in liver inflammation and tumorigenesis of glycogen storage disease type Ia mice receiving gene therapy. *Human Mol. Gene.* **2017**, *26*, 1890–1899, doi:10.1093/hmg/ddx097.
33. Lally, J.S.V.; Ghoshal, S.; DePeralta, D.K.; Moaven, O.; Wei, L.; Masia, R.; Erstad, D.J.; Fujiwara, N.; Leong, V.; Houde, V.P.; et al. Inhibition of Acetyl-CoA Carboxylase by Phosphorylation or the Inhibitor ND-654 Suppresses Lipogenesis and Hepatocellular Carcinoma. *Cell Metab.* **2019**, *29*, 174–182 e175, doi:10.1016/j.cmet.2018.08.020.
34. Wang, N.; Feng, Y.; Tan, H.Y.; Cheung, F.; Hong, M.; Lao, L.; Nagamatsu, T. Inhibition of eukaryotic elongation factor-2 confers to tumor suppression by a herbal formulation Huanglian-Jiedu decoction

in human hepatocellular carcinoma. *J. Ethnopharmacol.* **2015**, *164*, 309–318, doi:10.1016/j.jep.2015.02.025.

35. Bagga, S.; Rawat, S.; Ajenjo, M.; Bouchard, M.J. Hepatitis B virus (HBV) X protein-mediated regulation of hepatocyte metabolic pathways affects viral replication. *Virology* **2016**, *498*, 9–22, doi:10.1016/j.virol.2016.08.006.

© 2019 by the authors. Licensee MDPI, Basel, Switzerland. This article is an open access article distributed under the terms and conditions of the Creative Commons Attribution (CC BY) license (http://creativecommons.org/licenses/by/4.0/).
